# Religious Coping, Hopelessness, and Suicide Ideation in Subjects with First-Episode Major Depression: An Exploratory Study in the Real World Clinical Practice

**DOI:** 10.3390/brainsci10120912

**Published:** 2020-11-27

**Authors:** Domenico De Berardis, Luigi Olivieri, Gabriella Rapini, Nicola Serroni, Michele Fornaro, Alessandro Valchera, Alessandro Carano, Federica Vellante, Massimiliano Bustini, Gianluca Serafini, Maurizio Pompili, Antonio Ventriglio, Giampaolo Perna, Silvia Fraticelli, Giovanni Martinotti, Massimo Di Giannantonio

**Affiliations:** 1 Department of Mental Health, Psychiatric Service of Diagnosis and Treatment, Hospital G. Mazzini, NHS, ASL 4 Teramo, 64100 Teramo, Italy; gabriella.rapini@aslteramo.it (G.R.); nicola.serroni@aslteramo.it (N.S.); 2 Department of Neurosciences and Imaging, Chair of Psychiatry, University G. D’Annunzio, 66100 Chieti, Italy; federica.vellante@gmail.com (F.V.); silvieffesc@gmail.com (S.F.); giovanni.martinotti@gmail.com (G.M.); digiannantonio@unich.it (M.D.G.); 3 Department of Psychiatry, Federico II University, 80134 Naples, Italy; dott.fornaro@gmail.com; 4 Villa S. Giuseppe Hospital, Hermanas Hospitalarias, 63100 Ascoli Piceno, Italy; alessandrovalchera@gmail.com; 5 Department of Mental Health, Psychiatric Service of Diagnosis and Treatment, Hospital Madonna Del Soccorso, NHS, San Benedetto del Tronto, 63100 Ascoli Piceno, Italy; alessandro.carano@gmail.com; 6 Department of Mental Health, Psychiatric Service of Diagnosis and Treatment, Hospital San Camillo de Lellis, NHS, ASL Rieti, 02100 Rieti, Italy; m.bustini@asl.rieti.it; 7 Department of Neuroscience, Rehabilitation, Ophthalmology, Genetics, Maternal and Child Health, Section of Psychiatry, University of Genoa, 16126 Genoa, Italy; gianluca.serafini@unige.it; 8 Department of Neurosciences, Mental Health and Sensory Organs, Suicide Prevention Center, Sant’Andrea Hospital, Sapienza University of Rome, 00185 Rome, Italy; maurizio.pompili@uniroma1.it; 9 Department of Psychiatry, University of Foggia, 71122 Foggia, Italy; a.ventriglio@libero.it; 10 Department of Biomedical Sciences, Humanitas University, 20090 Milan, Italy; pernagp@gmail.com; 11 Department of Clinical Neurosciences, Villa San Benedetto Menni Hospital, Hermanas Hospitalarias, Albese Con Cassano, 22032 Como, Italy; 12 Department of Psychiatry and Neuropsychology, Faculty of Health, Medicine and Life Sciences, Maastricht University, 6200 MD Maastricht, The Netherlands; 13 Department of Psychiatry and Behavioral Sciences, Leonard Miller School of Medicine, Miami University, Miami, FL 33136, USA

**Keywords:** religiosity, religious coping, depression, suicide ideation, hopelessness, prevention

## Abstract

Background. This study aimed to evaluate the potential relationships between religious coping, hopelessness, and suicide ideation in adult outpatients with the first episode of major depressive disorder (MDD). Methods. Ninety-four adult outpatients with MDD were assessed through the Hamilton Depression Rating Scale (HAM-D), the Beck Hopelessness Scale (BHS), and the Scale of Suicide Ideation (SSI). Religious coping was assessed with the Italian version of the Brief RCOPE scale, consisting of seven positive coping items (PosCop) and seven negative coping items (NegCop). Results. The results showed that the Brief RCOPE PosCop scale exhibited a strong inverse correlation with HAM-D, BHS, and SSI, whereas HAM-D and BHS were positively correlated with SSI. Brief RCOPE NegCop scores were positively correlated only with SSI. Regression analysis with SSI as the dependent variable showed that higher Brief RCOPE PosCop scores were associated with lower suicide ideation, whereas higher HAM-D and BHS scores were associated with higher suicide ideation. Conclusion. Positive religious coping may be a protective factor against the development of suicide ideation, perhaps counteracting the severity of depressive symptoms and hopelessness. The evaluation of religious coping should be performed in all subjects with MDD in everyday clinical practice. However, this study was preliminary, and limitations must be considered.

## 1. Introduction

Major depressive disorder (MDD) is a disabling and common psychiatric disorder worldwide [1]. The relationships between MDD severity and suicide ideations have been investigated in several studies [2,3]. When associated, suicide ideation in MDD is a significant cause of the transition to suicidal behavior and attempts in such subjects, thus increasing the overall burden of the disorder [2,4].

Knowing the risk factors for suicide in MDD and how to get help can save lives [5,6]. Among these factors, hopelessness is often associated with an increase in suicide risk [7,8]. Hopelessness is a cognitive style characterized by pessimistic expectations for the future, absence of motivation, and the attribution of erroneous implications to own experiences [7,9]. It has also been suggested that hopelessness may act as a mediator between depressive symptoms and suicidality [10]. Actually, some authors pointed out that hopelessness may be even more informative than the presence of depressive symptoms in examining suicide ideation [11,12]. Even if the MDD severity may be a robust risk factor for suicide, both are likely to be related to hopelessness, which in turn could be a consequence of psychological pressures that result from lower social support and negative life events [12]. Moreover, despite MDD severity, higher psychache (i.e., an unbearable psychological pain) may be strongly related to hopelessness to explain suicidality [13,14]. However, some studies indicate that hopelessness is a risk factor for the short term (6 months) but not in long term [15,16].

Suicide prevention strategies are mandatory to reduce the phenomenon, and, even if the current literature is more focused on risk factors, the protective factors should always be taken into account [17,18].

One of these factors is religiosity [19,20]. It has been demonstrated that there is a negative association between religiosity and suicidality in the general population [21]. Moreover, a well-conducted meta-analysis on nine studies concerning religion and completed suicide found a generally protective influence of religiosity, with analyses revealing critical protective effects in Western cultures, nations with religious homogeneousness, and in the elderly [22]. The protective effects of religion regarding suicide are explained in several ways [23]. The frequent attendance at churches, the social networking and support within religious communities, the commitment to core religious beliefs, and the recourse to religion to effectively cope with negative events and life stressors, are all protective factors [20,24]. Interestingly, religiosity, either as general participation or through a religious association, is protective against suicide proportionally to the degree of participation in religious activities [25]. Moreover, moral and religious objections to suicide are associated with less suicidal behavior in depressed patients, and are suggested to act as protective factors against suicidal behavior [26,27].

To date, we recognize that faith, belief, and trust might reinforce psychological wellbeing [28,29]. Religious and spiritual beliefs and values may affect the course of several psychiatric disorders, including MDD [30,31]. Interestingly, Miller et al. [32] showed that a high self-report rating of religion and spirituality’s importance had a protective effect against the recurrence of MDD, particularly in adults with a history of parental MDD. For ten years, they followed longitudinally 114 adult offspring of depressed and nondepressed parents and found that those with the particular high significance of religion/spirituality had about one-fourth the risk of having an episode of depression over a 10-year prospective period [32].

The religiosity may enhance subjective wellbeing, thus lowering the impact of MDD [33]. Villani et al. [34] found that religious identity commitment positively predicted satisfaction with life among religious, but not among uncertain individuals. The social support given by religious communities, the feeling to be not alone but part of a close-knit group, the belief of a protective and forgiving God are also associated with higher subjective wellbeing [35,36].

Bergan et al. [37] defined religiosity as including “various dimensions associated with religious faiths and involvement.” Thus, religiosity denotes the variable tendencies to commit and adhere to religious beliefs, to follow religion principles, and attend activities and churches [38]. Nevertheless, while religiosity denotes religious participation as a whole, religious coping emphasizes how people rely on religion in a crisis state [39]. The positive religious coping (PRC) indicated “an expression of a sense of spirituality, a secure relationship with God, a belief that there is meaning to be found in life, and a sense of spiritual connectedness with others” [40]. The negative religious coping (NRC) is “an expression of a less secure relationship with God, a tenuous and ominous view of the world, and a religious struggle in the search for significance” [40].

Some studies have pointed out that religion may be even a risk factor for suicide in some cases [41,42]. Lawrence et al. [43] showed that previous suicide attempts were more frequent in MDD patients with a religious affiliation and suicide ideation was higher in MDD patients who considered religion very necessary, and those who regularly attended services. Huguelet et al. [44] pointed out that, although religion may be protective for several subjects with schizophrenia, someone occasionally wish to die and be with God, wish for another life after death, feel angry with God, or feel abandoned or unsupported by their religious communities. Moreover, a significant effect on suicide attempt history is the sensation of a punishment by God [45].

Overall, these evidences might be an indicator of an NRC [46]. Francis et al. [47] found that NRC, rather than PRC, demonstrated a significant association with depressive and anxiety symptoms, but they evaluated 622 medical students (therefore a nonclinical sample). Eskin et al. [48] found that NRC was significantly associated with suicide attempts in 7427 young adults affiliating with Islam, perhaps weakening religiosity’s protective effects. As well, Kopacz et al. [49] evaluated a sample of 772 recently returned veterans and showed that NRC was significantly associated with suicide risk, whereas no meaningful relationships for PRC were found. Trevino et al. [50] analyzed a sample of adult patients with advanced cancer and pointed out that NRC was associated with an increased risk for suicidal ideation, whereas PRC was not protective.

However, to date, no studies have assessed the relationships between religious coping, hopelessness, and suicide ideation in MDD [51,52]. Therefore, in the present exploratory study, we aimed to (i) evaluate the correlations between religious coping, hopelessness, and suicidal ideation in MDD, controlling for several variables; (ii) assess the effect of religious coping, hopelessness, and severity of MDD in the likelihood of suicidal ideation by using a linear regression analysis model.

## 2. Methods

A total of 94 adult outpatients (44 males, 50 females) of the Catholic faith with a Diagnostic and Statistical Manual for Mental Disorders, Fifth Edition (DSM-V) diagnosis of a first episode of MDD with melancholic characteristics were evaluated at several mental health facilities across Central Italy in a real world, everyday clinical practice setting from August 2019 to January 2020. Suitable outpatients satisfied the criteria for a Major Depressive Episode (MDE) with a score ≥18 on the Hamilton Depression Rating Scale (HAM-D), 17-item version [53]. The mean outpatient age was 25.3 ± 3.9 (age range 18–39 years) and were drug-naïve treatment seekers for MDD. We focused on drug-naïve outpatients as they were not under the influence of pharmacotherapy or psychotherapy that affects the feelings and thoughts about religiosity. The mean duration of illness was 5.7 ± 3.0 months.

The exclusion criteria were as follows: any other axis-I and II disorder, any substance use disorder, intellectual disability, severe medical diseases, and the existence of any organic mental disorders.

Religious coping was assessed with the Italian version of the Brief RCOPE scale [54,55]. The Brief RCOPE is a self-report scale and comprises seven positive coping items (PosCop) and seven negative coping items (NegCop). The seven PosCop items are spiritual connection, seeking spiritual support, religious forgiveness, collaborative religious coping, benevolent religious reappraisal, religious purification, and religious focus. The seven NegCop items are spiritual discontent, punishing God reappraisal, interpersonal religious discontent, demonic reappraisal, and reappraisal of God’s power. A 4-point Likert scale with answer choices from one (strongly disagree) to four (strongly agree) is used to respond to all the 14 items. Both Brief RCOPE scales range from seven (low) to twenty-eight (high). The mean scores of PosCop and NegCop were respectively 19.1 ± 5.8 and 12.9 ± 3.8.

The Scale of Suicide Ideation (SSI) was administered to objectively evaluate the presence of a suicidal ideation [56]. The SSI is one of the most commonly used rating scales and consists of a 19-item, interviewer-administered rating scale. Each item comprises three choices classified according to the strength of the suicidal ideation and it is assessed on a three-point scale ranging from 0 to 2 (not at all ideation to robust ideation). The total score is considered and it ranges from 0 to 38. The mean score of SSI was 10.8 ± 6.1.

The Beck Hopelessness Scale (BHS), a 20-item scale, was utilized to measure the hopelessness feelings and the negative attitudes about the future [57]. The BHS consists of 20 “true/false” items that target the main characteristics of hopelessness. Higher overall scores designate more hopelessness (range 0–20). The mean score of BHS was 13.0 ± 3.6.

The severity of MDD was calculated with the 17-item HAM-D total score [53]. The HAM-D is one of the most used interviewer-administered rating scales in the evaluation of depressive symptoms. The total score is obtained by the sum of the single scores from each item. The mean score of HAM-D was 28.3 ± 4.0.

All the rating scale records were collected during the real world, routine clinical practice and patients’ assessment and, consequently, no institutional review board authorization was needed. Nevertheless, each patient had to comprehend the study’s characteristics and gave us a written informed consent before enrollment.

## 3. Statistical Analysis

Partial correlations between Brief RCOPE subscales, BHS, HAM-D, and SSI controlling for age, gender, and duration of illness were used. A blockwise linear regression analysis was executed to determine which variables were associated with the severity of suicidal ideation (SSI as a dependent variable). Age, gender, and duration of illness were added to the first block. At the second block, BHS and HAM-D scores were added to the model. The Brief RCOPE subscales scores were entered in the third and last step. A more conservative value of *p* ≤ 0.01 was considered statistically significant. All statistical testing was two-tailed.

## 4. Results

Gender comparisons between demographic and clinical variables showed no significant differences concerning any of the accounted variables. Partial correlations between Brief RCOPE subscales, BHS, HAM-D, and SSI total scores controlling for age, gender, and duration of illness showed that Brief RCOPE PosCop scores were negatively correlated with BHS (*r* = −0.43, *p* < 0.001), HAM-D (*r* = −0.34, *p* = 0.001), and SSI (*r* = −0.57, *p* < 0.001). Brief RCOPE NegCop scores were positively correlated only with SSI (*r* = −0.26, *p* = 0.01). In contrast, HAM-D was positively correlated with both BHS (*r* = 0.35, *p* = 0.001) and SSI (*r* = 0.45, *p* < 0.001). BHS and SSI scores were positively correlated (*r* = 0.55, *p* < 0.001).

In the linear regression models (Table 1), higher Brief RCOPE PosCop scores were associated with lower suicide ideation (using SSI as a dependent variable). In contrast, higher BHS and HAM-D scores were associated with higher suicide ideation. In the current analyses, the *R*^2^ values accounted for 51% of the SSI score variance. In addition, the Durbin–Watson coefficient was 1.798 (near to the optimum of 2.0). A scatter plot of residuals and a plot of regression-standardized residuals indicated a near-normal distribution.

## 5. Discussion

To our knowledge, this was the first study that evaluated the relationships between religious coping, hopelessness, and suicidal ideation in a sample of outpatients with MDD in a real world setting of everyday psychiatric practice.

Patients with a higher PRC showed less MDD severity, and these results may account for a potential protective effect of religiosity in the development and course of MDD. We hypothesize that a PRC may stimulate the resilience in several persons at high risk of developing MDD, thus increasing the possibility of coping better with the disorder [58]. The PRC may enhance subjective sense of belonging, thus reducing the feeling of loneliness and alleviating depressive symptoms, often with the help of the social support given by other religious affiliates [59]. On the other hand, there is evidence that the personal distress associated with MDD might lead to increased religious involvement in order to receive support, solace, and help, and this may be true also for our studied sample [60]. Moreover, the observed high PRC in our study may give the person a better sense and a functional meaning to his/her own life, reducing the impact of severity of MDD [61].

The present study’s main results were that PRC was associated with less severe suicidal ideation and hopelessness. It was a predictive factor of reduced suicide risk in a linear regression model, somewhat counteracting MDD severity and the prosuicidality effect of hopelessness.

However, religious coping would be protective or even disadvantageous depending on the way of coping [62]. In several studies, PRC was not a protective factor of suicide, whereas NRC was associated with a higher risk of suicide [48]. However, our study found that NRC correlated positively with higher suicide ideation, but this association was not present in the logistic regression model. Therefore, religious coping seems to be an ambivalent feature that may be linked to increased suicide ideation when a there is a prevalence of a negative coping. [63]. The results of our study point out that a positive coping may counteract the negative attitudes, leading to reduced severity of depression and hopelessness, thus minimizing NRC’s detrimental effects. Our study results may also be explained considering that our sample was composed of Catholics and therefore, the characteristics of this religion may have an importance. Eskin et al. [64] reported that the affiliation with Catholicism and Orthodox Christianity was associated with decreased risk for psychological distress. We argue that the associations of the strength of religious belief against suicidal ideation have a positive relation in persons affiliating with Catholicism [65].

Moreover, the mean scores of PRC and NRC on Brief RCOPE in our sample were 19.1 ± 5.8 and 12.9 ± 3.8, respectively. This may suggest that our sample showed a tendency toward a PRC. This may explain why our results contrast with those that demonstrated the inability for PRC to reverse negative effects of NRC. The relative higher PRC observed in our sample also probably explains the observed lower hopelessness.

The presence of a PRC may have several positive consequences in MDD subjects. Positive religious coping could help MDD subjects to alleviate the stress, preserve a sense of control, and sustain hope and self-esteem, thus giving significance to life even in an acute depressive episode. Rosmarin et al. [66] conducted a prospective study in a clinical sample of 47 psychiatric patients with current/past psychosis receiving day treatment at McLean Hospital. They found that PRC was associated with significantly more significant reductions in depression and anxiety, and increases in wellbeing throughout treatment.

In our study, the lesser intensity of hopelessness may also mediate PRC positive effects. Mihaljevic et al. [67] measured hopelessness and religious coping style in 111 Croatian war veterans with Post-Traumatic Stress Disorder (PTSD) vs. 35 controls. In the PTSD group, less hopeless veterans showed more extensive use of PRC strategies. Regarding PRC’s positive effect, another possible explanation of our findings is that in previous studies, the evaluated samples were mixed concerning psychiatric diagnoses and did not concentrate only on MDD subjects as we did in our study.

Several theories may explain the lower risk of suicidality in persons with a PRC and religious affiliation [68]. First, coping with personal problems may be alleviated by relating oneself with God, spiritual supervision, and faith principles [69,70]. Moreover, religiosity’s protective effect on suicidality may also be mediated by Catholic communities’ strong social support to the subject with MDD [71]. The individuals with religion may be more likely to have more robust social support and less likely to be utterly isolated than those without religion [72]. These factors may be highly protective against the MDD-related suicide risk [73]. Jacob et al. [21] evaluated religiosity’s association with suicidal ideation and suicide attempts in the United Kingdom nationally representative sample using cross-sectional data from 7403 people who participated in the 2007 Adult Psychiatric Morbidity Survey. After controlling for several social and psychopathological variables, they found a significant and negative association of religiosity with past 12-month suicidal ideation, lifetime suicidal ideation, and lifetime suicide attempts. More recently, Gawad et al. [74] evaluated 688 adults admitted to an acute psychiatric facility with a primary mood or psychotic disorder and found that high religiosity scores were associated with significantly less suicidal ideation.

The finding of our study can be explained also considering that PRC is significantly associated with intrinsic religiousness. As noted by Masters [75], intrinsic religiousness is characterized as religion being a main motivation and people view the religion as the basis for their lives, trying to constantly live the religion they believe (“My whole approach to life is based upon my religion” [75]). Intrinsic religiousness has been associated with faster remission of MDD, lower suicide risk, and better outcomes among MDD patients [76]. Smith et al. [77] found that intrinsic religiousness was a measure of “positive” religiousness and had stronger negative correlations with symptoms of depression. PRC is related to intrinsic religiousness and has a protective function at a high level of controllable negative events [78]. Mosquero et al. [79] showed that intrinsic religiousness was associated with higher resilience, better quality of life, and fewer previous suicide attempts in MDD patients. The protective effects of intrinsic religiousness are mediated by positive attitudes toward religion [80]. Interestingly, higher serum brain-derived neurotrophic factor levels were found in MDD patients with higher intrinsic religiousness, and this may be one of the neurobiological explanations of positive effects of PRC in MDD and suicidal ideation [81]. We can argue that intrinsic religiousness and PRC enhance personal resilience at several levels, thus reducing the severity of depression and the emergence of suicidal ideation and behaviors [79,82]. On the other hand, NRC is associated with the extrinsic religiousness (i.e., the use of religion for a variety of personal needs such as security, status, or social support) and this is considered as a potential risk factor for MDD, lower self-control, and suicide [83,84]. Extrinsic religiousness is also related to a lower psychological wellbeing, probably through a perceived negative attitude toward religion [85]. Moreover, Masters and Knestel [86] found that people with extrinsic religiousness were more likely to be divorced and reported a general poorer health, a higher body mass index, and a greater use of tobacco and alcohol than those with intrinsic religiousness.

Nevertheless, it has been demonstrated that religiosity, either as broad participation or through a religious association, was associated with protection against suicide proportional to the degree of participation in religious activities [25]. Jongkind et al. [46] demonstrated that higher moral objections to suicide (MOS) and a positive-supportive God representation in Catholic patients with MDD were negatively correlated with suicide ideation. This may be putatively true for our sample which consists of Catholics, but we did not assess the degree of participation in religious activities.

## 6. Limitations

The present study was preliminary and exploratory. As such, we would prompt the following limitations in the interpretation of these preliminary results.

The first limitation was the relatively small sample size (even if all the evaluated patients were drug naïve, who are often hard to find and evaluate in everyday clinical practice). Additionally, even if the severity of MDD and suicidal ideation were analyzed using clinician-rated rating scales, religious coping and hopelessness were assessed by self-rated scales, with probable biases due to the intrinsic nature of self-rating scales. Moreover, the partial correlation analyses were controlled for three clinical variables, thus making it potentially less reliable. In addition, we did not assess the degree of participation in religious activities that would be a variable of importance.

Furthermore, we employed a cross-sectional design that limits statements regarding causality—our study lacks follow-up data. The present study’s cross-sectional method precluded any firm conclusion about any eventual hierarchical role interaction of either religious coping or hopelessness or MDD severity in determining suicide risk.

## 7. Conclusions

In conclusion, our study’s findings lean towards PRC’s potential protective role against the development of suicide ideation, thus reducing the suicide risk in MDD subjects. This positive action of PRC is probably exerted through a counteraction of prosuicidal effects of hopelessness and depression severity and NRC’s attenuation.

We firmly believe that discussing religiosity with MDD subjects may offer insights into why some people are more likely to consider or not suicide, which may have implications for both patient care and the institution of effective suicide prevention strategies [24,87]. In several cases, the collaboration with spiritual care providers, such as priests and ecclesiastics, may enhance suicide prevention efforts [45,49].

The subject’s spiritual requests should be addressed early when diagnosing an MD episode. We also advise that religiosity should always be measured in the real world, everyday clinical practice in the subjects with MDD. In a very interesting and comprehensive review, Gearing and Alonzo [23] wrote that “the relationship between an individual’s religiosity and suicidality is often minimized or ignored in clinical assessments…” and this is sad, but true. They suggest several general practice recommendations for clinicians who assess and try to understand the effect and impact of patients’ religiosity on their suicide risk [23]. The availability of rating scales is undoubtedly a great help for the assessment the different facets of religion in real world, everyday clinical practice, and these should be routinely used. On the basis of our study results, we believe that it is necessary to evaluate the religious coping strategies, and, therefore, the use of Brief RCOPE in real world, everyday clinical practice is very brief, straightforward, and recommended.

Even if pharmacological therapy is mandatory in severe MD, the therapies should also strengthen the PRC patterns of subjects and discover a possible recourse to NRC that may destructively affect the outcome. Thus, a continuous assessment of the patients’ religious needs could be appropriate, but further prospective studies are needed.

## Figures and Tables

**Table 1:** Results of the linear regression analysis with Scale of Suicide Ideation (SSI) as the dependent variable and other variables as independents.

Model		Unstandardized Coefficients		Standardized Coefficient	*t*	*p*	95% Confidence Interval for B	
		B	SE	Beta			Lower Bound	Upper Bound
Block 1	(Constant)	6.06	4.30		1.41	0.16	−2.49	14.61
	Age	0.11	0.16	0.07	0.69	0.49	−0.21	0.43
	Gender	−1.09	1.25	−0.09	−0.87	0.39	−3.58	1.40
	Duration	0.44	0.21	0.22	2.09	0.04	0.02	0.86
Block 2	(Constant)	−14.29	4.74		−3.01	0.00	−23.72	−4.86
	Age	0.09	0.13	0.06	0.67	0.50	−0.17	0.34
	Gender	−0.45	1.01	−0.04	−0.45	0.66	−2.45	1.55
	Duration	0.17	0.17	0.08	0.96	0.34	−0.18	0.51
	BHS	0.74	0.15	0.44	4.98	0.00	0.44	1.03
	HAM-D	0.45	0.14	0.30	3.27	0.00	0.18	0.72
Block 3	(Constant)	−1.41	5.71		−0.25	0.81	−12.76	9.93
	Age	0.04	0.12	0.02	0.31	0.75	−0.20	0.27
	Gender	−0.27	0.94	−0.02	−0.28	0.78	−2.13	1.60
	Duration	0.17	0.16	0.09	1.09	0.28	−0.14	0.49
	BHS	0.50	0.15	0.30	3.38	0.00	0.20	0.79
	HAM-D	0.32	0.13	0.21	2.49	0.01	0.07	0.58
	PosCop	−0.37	0.09	−0.35	−3.99	0.00	−0.55	−0.18
	NegCop	0.14	0.12	0.09	1.15	0.25	−0.10	0.39

Block 1. *R*^2^ = 0.05, F = 1.7, dF = 93, *p* < 0.18. Block 2. *R*^2^ = 0.41, F = 12.3, dF = 93, *p* < 0.001. Block 3. *R*^2^ = 0.51, F = 12.9, dF = 93, *p* < 0.001. Abbreviations: SE—Standard Error; BHS—Beck Hopelessness Scale; HAM-D—Hamilton Depression Rating Scale; PosCop—Positive Coping Subscale of Brief RCOPE; NegCop—Negative Coping Subscale of Brief RCOPE.
